# Asymptomatic pediatric presentation of S‐adenosylhomocysteine hydrolase deficiency

**DOI:** 10.1002/jmd2.12449

**Published:** 2024-09-15

**Authors:** Patrícia Lipari Pinto, Marjorie Dixon, Sniya Sudhakar, Ivo Baric, Julien Baruteau

**Affiliations:** ^1^ Hereditary Metabolic Disease Reference Center, Metabolic Unit, Pediatric Department Santa Maria's Hospital‐Lisbon North University Hospital Center, EPE, Pediatric University Clinic, Faculty of Medicine, University of Lisbon Lisbon Portugal; ^2^ Dietetics, Great Ormond Street Hospital for Children NHS Foundation Trust London UK; ^3^ Department of Radiology Great Ormond Street Hospital for Children NHS Foundation Trust London UK; ^4^ Department of Pediatrics University Hospital Center Zagreb and University of Zagreb, School of Medicine Zagreb Zagreb Croatia; ^5^ Department of Paediatric Metabolic Medicine Great Ormond Street Hospital for Children NHS Foundation Trust London UK; ^6^ National Institute of Health Research Great Ormond Street Biomedical Research Centre London UK; ^7^ Great Ormond Street Institute of Child Health, University college London London UK

**Keywords:** *AHCY* gene, hepatocellular carcinoma, hypermethioninemia, S‐adenosylhomocysteine, S‐adenosylhomocysteine hydrolase deficiency, S‐adenosylmethionine

## Abstract

S‐adenosylhomocysteine hydrolase deficiency is an autosomal recessive inborn error of metabolism affecting methylation by disrupting the methionine cycle. Its clinical spectrum spans from severe perinatal encephalomyopathy and liver failure to asymptomatic course in patients with isolated hypermethioninemia. We present two new cases of S‐adenosylhomocysteine hydrolase deficiency from Pakistani origin clinically asymptomatic at presentation. Both siblings showed mild chronic liver failure and elevation of creatine kinase. The older patient presented at 6 years of age with isolated verbal processing difficulty and mild diffuse leukodystrophy, reversible 12 months after introduction of methionine dietary restriction. The patient showed subtle atrophy in the muscle MRI at the age of 7 years. S‐adenosylhomocysteine hydrolase deficiency was confirmed with homozygous missense variant c.146G>A (p.Arg49His) in the *AHCY* gene, a genotype previously reported in Pakistani patients with mild presentation. Dietary methionine restriction decreased plasma methionine but not plasma S‐adenosylhomocysteine and S‐adenosylmethionine. This work expands the mild spectrum of S‐adenosylhomocysteine hydrolase deficiency with no noticeable clinical symptoms in children, highlighting a specific hotspot variant from South Asia. This mild form of the disease is likely underdiagnosed and raises the question of therapeutic management to prevent long‐term complications documented in the literature, such as hepatocellular carcinoma and myopathy in early adulthood.

**Synopsis**

1. Our study unveils a genotype‐phenotype correlation between in patients with S‐adenosylhomocysteine hydrolase deficiency. This aspect is pivotal for medical practitioners when predicting disease progression and deciding on appropriate interventions.
2. We have identified a likely high frequency variant associated with this condition within the Pakistani population. This finding emphasises the importance of region‐specific genetic screenings and counselling, which could lead to early diagnosis and better patient outcomes.
3. Our research underscores the challenges of maintaining the prescribed diet for affected however asymptomatic individuals.
4. An unprecedented discovery in our study was the noticeable response of leukodystrophy to the prescribed diet in one of the patients. This observation expands the current understanding of the disease and shows the importance of maintaining the diet even in asymptomatic patients.
5. Our research has confirmed the presence of specific muscular dystrophy in one of our study patients through advanced imaging techniques. This finding emphasises the varied manifestations of the disease and highlights the importance of comprehensive diagnostics.

## 1 INTRODUCTION

**FIGURE 1:**
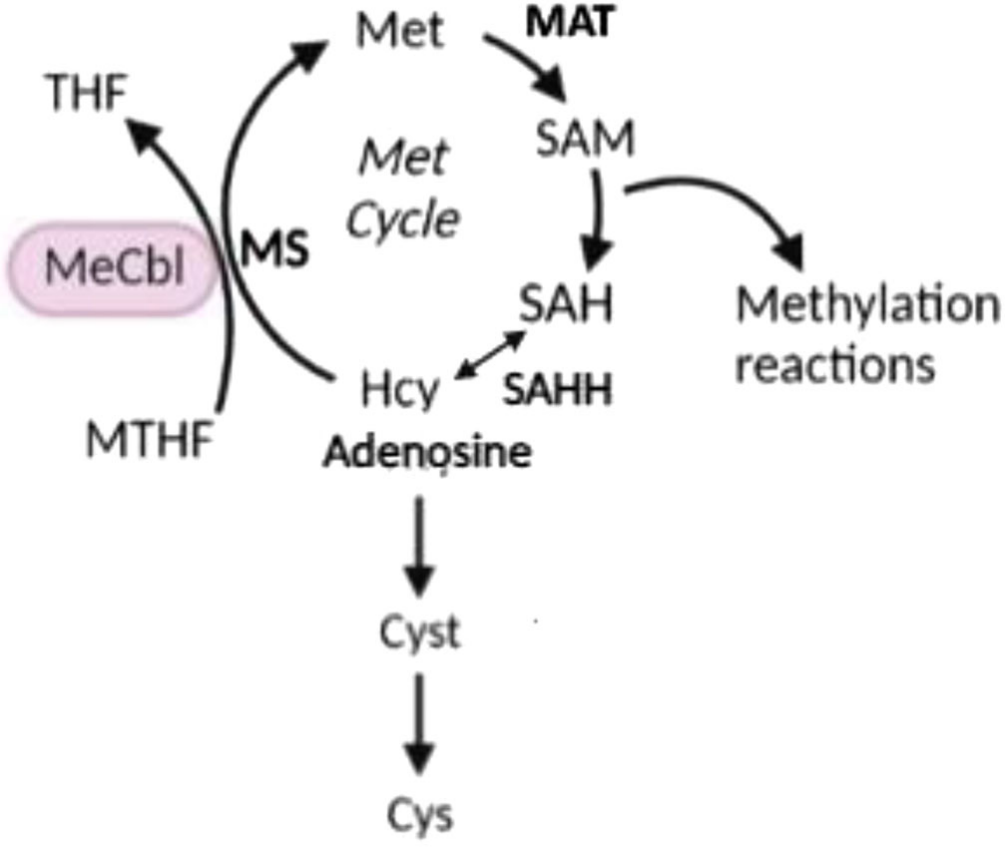
Schematic of the methylation cycle. Cys, cysteine; Cyst, cystathionine; Hcy, homocysteine; MAT, methionine adenosyltransferase; MeCbl, methylcobalamin; Met cycle, methionine cycle; MS, methionine synthase; MTHF, N5‐methyltetrahydrofolate; SAH, S‐adenosylhomocysteine; SAHH, S‐adenosylhomocysteine hydrolase; SAM, S‐adenosylmethionine.

Inherited methylation disorders are rare diseases affecting transmethylation reactions from methionine to homocysteine.^1^ Methylation disorders predominantly affect the central nervous system, liver, and skeletal muscle, with a large spectrum of clinical severity.^1^ Isolated hypermethioninemia, although not always present, is the biochemical hallmark of these disorders; in plasma amino acid chromatography, plasma S‐adenosylmethionine (SAM), and S‐adenosylhomocysteine (SAH) are key metabolites for assessing the enzymatic deficit.^1^ S‐adenosylhomocysteine hydrolase (SAHH) catalyzes the hydrolysis of SAH to adenosine and homocysteine, the only endogenous source of homocysteine in mammals.^2^ SAH is formed as a product of transmethylation reactions.^2^ SAM is the primary methyl donor for delivering methyl groups to DNA, RNA, proteins, and cellular metabolites (Figure 1).^2^, ^3^, ^4^ SAM/SAH balance plays a critical role in methionine metabolism and regulating gene expression through methylation.^2^ Aberrant methylation processes resulting from the significant imbalance in the SAM/SAH ratio are supposed to play a substantial role in the pathophysiology of the SAHH deficiency.^2^, ^5^

The clinical description of SAHH deficiency (SAHHD) was first reported in 1979 in a seven‐year‐old girl with severe myopathy, developmental delay, and hypermethioninemia.^6^ The first confirmed case of SAHHD was identified by Barić et al. in a Croatian boy in 2004.^7^ Pathogenic variants in the *AHCY* gene, which encodes the SAHH enzyme, causes hypermethioninemia, increased SAM and SAH with a significant imbalance in SAM/SAH ratio. Sixteen patients have now been reported. Due to the rarity of this disorder, the natural history and the clinical spectrum remain unclear. In most cases, severe phenotype with encephalopathy and myopathy is reported in infancy or early childhood.^7^, ^8^, ^9^, ^10^, ^11^, ^12^, ^13^, ^14^, ^15^ However, Stender et al.^14^ have also identified a mild late‐onset phenotype.

Here, we report two siblings with mild late‐onset SAHHD, incidentally diagnosed due to asymptomatic elevation of aminotransferases, elevation of CK, and hypermethioninemia, highlighting a genotype–phenotype correlation with previously reported late‐onset cases.^14^ Brain MRI showed a reversible leukodystrophy with the implementation of a methionine‐restricted diet. Dietary management normalized methioninemia but did not improve liver or muscle biomarkers or plasma SAH and SAM levels.

## 2 PATIENTS AND METHODS

### 2.1 Ethics

This study was conducted following the ethical standards of the Declaration of Helsinki and was approved by the National Research Ethics Service Committee London‐Bloomsbury (13/LO/0168). Written consent was obtained for both siblings.

### 2.2 Data collection

We retrospectively collected clinical, biochemical, and neuroradiological data from medical records with an analysis of dietary management and clinical outcome.

### 2.3 Metabolite measurements and genetic sequencing

Serum amino acids and homocysteine in plasma, CK, clotting screen, and aminotransferases were measured using standard biochemical methods at Great Ormond Street Hospital for Children (GOSH), London, UK. Dried blood spots were measured by the Metabolic lab, Guys, and St Thomas Hospital, London, UK.

SAH and SAM levels and genetic sequencing of the *AHCY* gene were performed at the VU Medical Centre, Clinical Chemistry—Metabolic Laboratory, from the University of Medicine of Amsterdam (UMC), The Netherlands.

### 2.4 Imaging

Muscle and brain MRI, transthoracic echocardiogram, and liver and portal system ultrasound with Doppler were performed according to standard procedure at GOSH, London, UK.

### 2.5 Literature search

A literature review was performed in PubMed for SAHHD. Medical subject heading (MeSH) terms used in the literature search included “S‐Adenosylhomocysteine hydrolase deficiency,” “SAH hydrolase deficiency,” “SAHH deficiency,” and “AHCY variant.”

## 3 RESULTS

### 3.1 Patient report

#### 3.1.1 Patient 1

##### Clinical presentation

The first child is a female born at 37 weeks of gestation by ventouse delivery from consanguineous parents from Pakistan. Pregnancy and delivery were uneventful. Growth parameters were normal. Early developmental milestones were age‐appropriate in all domains, although she tired quickly when she first started to walk.

**FIGURE 2:**
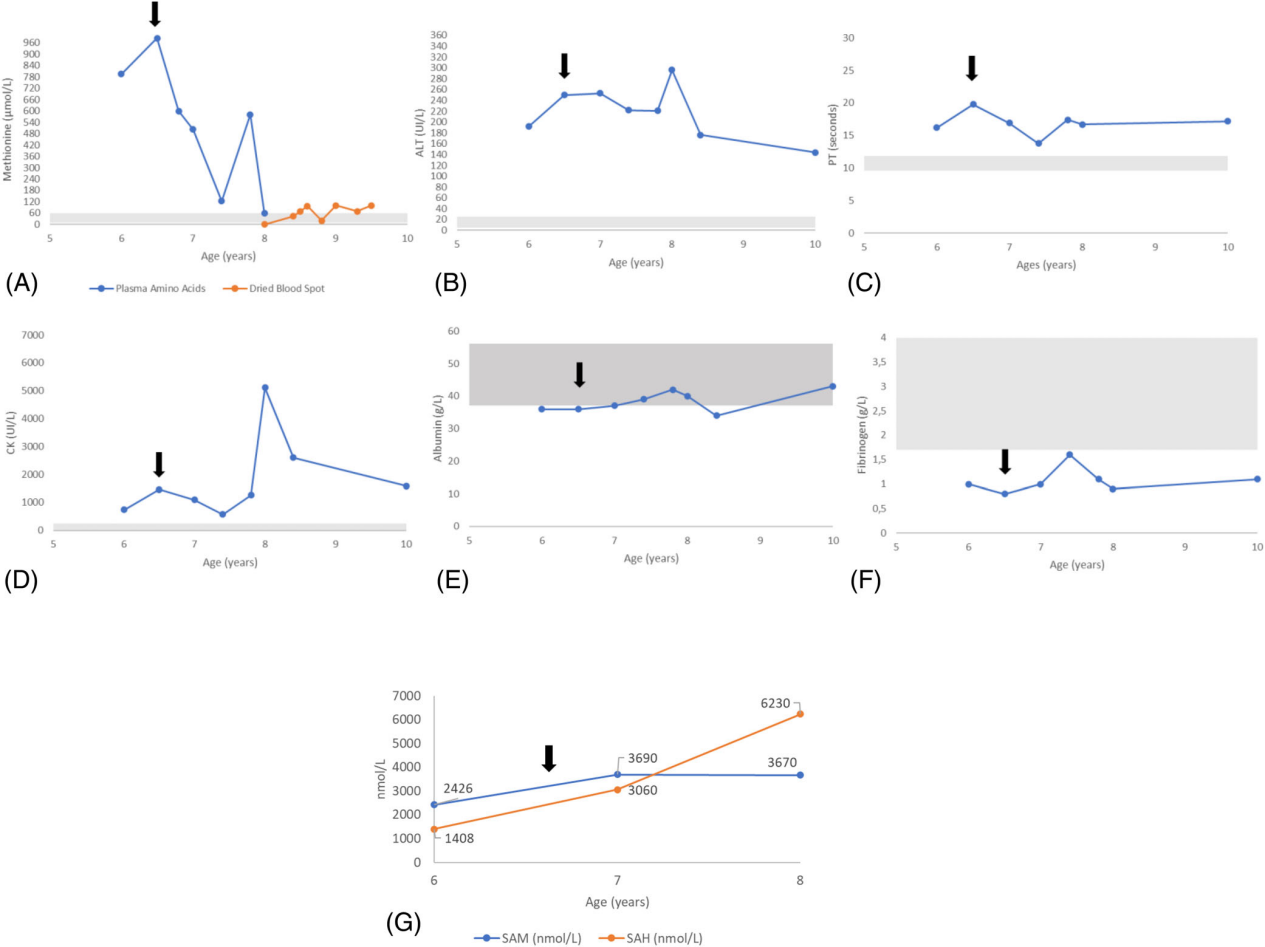
Biochemical profiles of patient 1. (A) Methionine, (B) ALT, (C) PT, (D) CK, (E) Albumin, (F) Fibrinogen, and (G) SAM and SAH, the dark arrow represents commencement of methionine‐restricted diet. The gray‐shaded area in each graphic represents the normal reference range values. ALT, alanine aminotransferase; CK, creatine kinase; PT, prothrombin time; SAH, S‐adenosylhomocysteine; SAM, S‐adenosylmethionine.

At the age of five, she developed viral gastroenteritis. Subsequent blood tests revealed her ALT level was six times higher than the normal range (Figure 2). Subsequent ALT monitoring showed a persisting increase after the resolution of the intercurrent illness, triggering investigations for infectious, autoimmune, and metabolic etiologies. This revealed isolated plasma hypermethioninemia at 985 μmol/L (normal 10–60) and high urine methionine/creatinine ratio at 46 μmol/mmol (normal 3–10), mildly elevated plasma homocysteine level 15 μmol/L (normal 5–10), and elevation of muscle enzymes with elevated ALT 250 IU/L (normal 5–25) and high CK 1450 IU/L (normal <250). Indicators of chronic liver failure were observed, with mildly low albumin level 36 g/L (normal 37–56) and a normal serum alpha‐fetoprotein (AFP) level. Notably, coagulation abnormalities, which are frequently seen in SAHH deficiency, were also present, as evidenced by a prolonged PT of 19.8 s (normal 9.6–11.8) and low fibrinogen levels of 0.8 g/L (normal 1.7–4.0) (Figure 2). Despite these biological abnormalities, the clinical examination was unremarkable. Specifically, there were no clinical signs suggesting coagulation abnormalities. Additionally, there was an absence of visceromegaly, normal muscular strength (including a negative Gowers test), and a standard neurological examination. A formal neuropsychological assessment using the Wechsler Intelligence Scales for Children (WISC‐V) showed good motivation, concentration, and intellectual abilities in the low average range. However, she produced an uneven profile, with weaker abilities in verbal reasoning abilities (below average) and strengths in processing speed (high average). The child attends mainstream school with no difficulties with academic tasks and physical exercise at age 9. Growth was appropriate with weight on the 9th centile and height on the 25th centile.

##### Diagnostic tests/imaging

**FIGURE 3:**
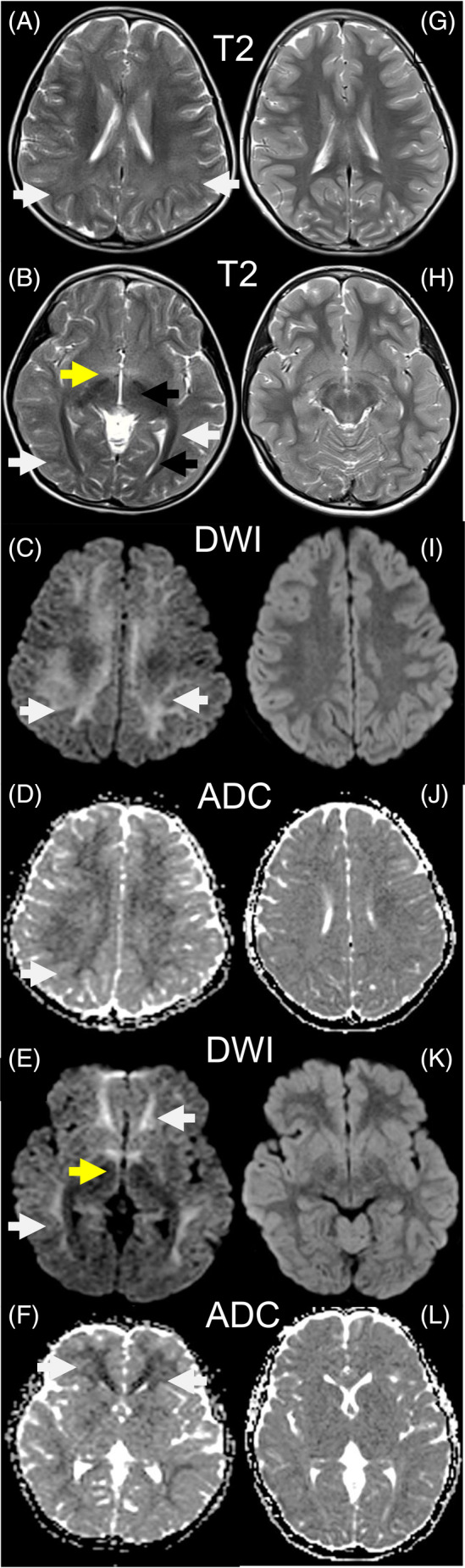
Neuroimaging of patient 1. (A–F) A brain MRI from patient one performed at the age of 6.5 years before commencement of methionine‐restricted diet shows extensive and confluent hypersignal changes of deep white matter involving all the lobes (A, B; white arrows), and the anterior commissure is also engaged (B; yellow arrow) in T2 sequences. There is corresponding diffusion restriction on diffusion‐weighted imaging (DWI) (C, D) and apparent diffusion coefficient (ADC) (E, F) sequences. The optic radiation and internal capsule are spared (B; black arrows). (G–L) The brain MRI at 7.5 years (after 12 months of methionine‐restricted diet) shows resolution of all white matter changes without residual volume loss or signal change.

12 leads ECG, echocardiography, and liver ultrasound were normal. Her brain MRI showed mild leukodystrophy with confluent hyperintense white matter signal changes predominantly involving the deep and subcortical white matter, sparing the extreme subcortical, periventricular, and parts of deep white matter (Figure 3). A muscle MRI showed only a minimal bilateral streaking of the posterior compartment muscles, especially involving the adductor Magnus.

##### Diagnosis

SAM and SAH showed a massive increase, with SAM 2426 nmol/L (normal 55–116) and SAH 1408 nmol/L (normal 9–45). The sequencing of the *AHCY* gene, which encodes SAHH, identified homozygous missense pathogenic variant c.146G>A (p.Arg49His), confirming the diagnosis of SAHH deficiency. Parental segregation analysis was performed, and both parents were found to be heterozygous for the variant.

##### Therapeutic management

A methionine‐restricted diet was initiated at 6 years old and has been followed for 2 years. In the United Kingdom, due to the limited amino acid analysis available for foods, it was challenging to restrict methionine intake directly. As a result, the approach focused on reducing natural protein consumption to limit methionine indirectly. It's estimated that 1 g of protein yields approximately 20 mg of methionine. The primary goal of the dietary modification was to lower plasma methionine levels to within the normal reference range. The literature suggests that methionine intakes ranging from 15 to 40 mg/kg/day effectively reduce plasma methionine to low or normal levels, as noted in studies by Baric and Huang.^7^, ^9^, ^12^, ^16^ It is important to note that the World Health Organization (WHO) recommends a minimum safe methionine intake of 15–20 mg/kg/day for infants and young children, corresponding to a protein intake of 0.75–1 g/kg/day.^16^ However, this guideline lacks direct referencing and may require further substantiation. In this case, dietary intervention gradually reduced protein consumption. Initially, the patient's diet contained an estimated 50 g of protein per day (2.8 g/kg/day). Over a period of 6 months, this was reduced to align with the FAO/WHO/UNU 2007 guidelines for a safe level of protein intake for the patient's age, which is 0.91 g/kg/day (equating to 18 mg of methionine/kg/day). Additionally, a methionine‐free, cystine‐enriched amino acid supplement was introduced. This supplement was gradually increased from 0.5 to 1.0 g/kg/day of protein equivalent over the first 6 months and further to 1.5 g/kg/day from the ninth month onward. This increase ensured an adequate intake of all other essential amino acids, vitamins, and minerals. The dietary regimen was monitored through regular measurements of plasma or blood spot methionine levels (Figure 2, for details).

It's noteworthy that adhering to this dietary restriction, along with the provision of a full dose of amino acid supplement and the necessity of monthly blood spots for methionine monitoring, posed significant challenges for the patient and their parents. These interventions were not always feasible to implement consistently.

The family opted against therapy involving creatine, phosphatidylcholine, and N‐acetylcysteine.

##### Evolution

The child remained clinically stable with no academic limitations and normal physical status. The diet enabled a significant reduction of methionine levels despite difficulties with adherence (Figure 2, graph A). However, this did not modify SAM, SAH, CK, clotting parameters, and liver transaminase plasma levels (Figure 2, graphs B–G). After 12 months of a methionine‐restricted diet, a complete resolution of the previous changes in her brain MRI was observed (Figure 3).

#### 3.1.2 Patient 2

##### Clinical presentation

Patient 2 is the younger brother of the proband patient 1. He was born at term by normal delivery after an uneventful pregnancy. Growth parameters were within the normal ranges. There was no neonatal concern. Early developmental milestones were age‐appropriate. There have been no significant health concerns.

A familial screening following his sister's diagnosis resulted in a diagnosis at the age of 13 months. His clinical examination was unremarkable, with no organomegaly, normal neurological examination, and muscular strength. Growth was appropriate with weight tracking to 25–50th centile and height on the 50th centile.

**FIGURE 4:**
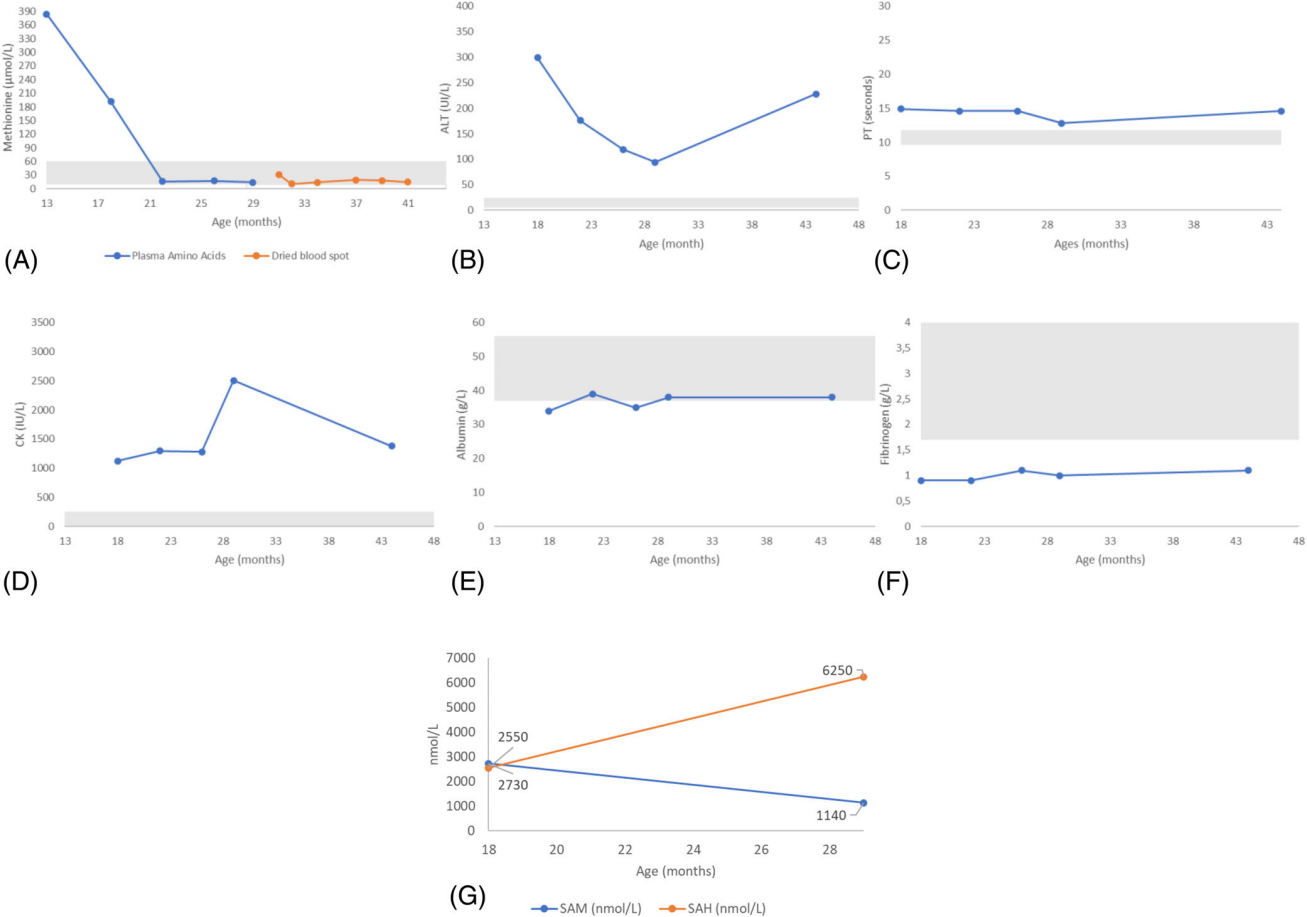
Biochemical profiles of patient 2. (A) Plasma methionine, (B) ALT, (C) PT, (D) CK, (E) Albumin, (F) Fibrinogen, (G) SAM and SAH. The gray‐shaded area in each graphic represents the normal reference range values. ALT, alanine aminotransferase; CK, creatine kinase; PT, prothrombin time; SAH, S‐adenosylhomocysteine; SAM, S‐adenosylmethionine.

Biological findings in the brother were similar to his sister's. They include isolated plasma hypermethioninemia at 384 μmol/L (normal 10–60), mildly elevated total homocysteine level 14 μmol /L, (normal 5–10), and chronic liver failure with elevated ALT 250 IU/L, (normal 5–30), and potential coagulation abnormalities, suggested by prolonged PT 14.9 sec (normal 9.6–11.8) and low fibrinogen 0.9 g/L (normal 1.7–4.0). The elevated ALT could also be related to muscle involvement, as evidenced by a high CK 1125 IU/L (normal <250). Additionally, he had elevated SAM 2730 nmol/L (normal 55–116) and SAH 2550 nmol/L (normal 9–45) levels (Figure 4, graph G). AFP was within the normal range.

##### Imaging

**FIGURE 5:**
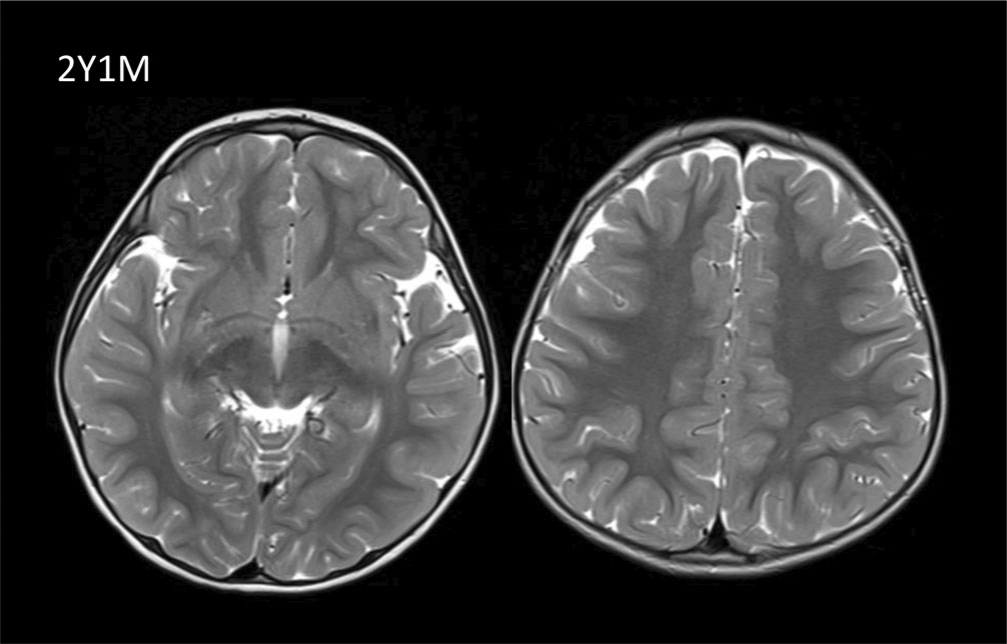
Neuroimaging of patient 2. Normal MRI at 2 years of age.

At 26 months of age, after 12 months of a methionine‐restricted diet, a brain MRI was normal (Figure 5). Concomitantly, a muscle MRI from the pelvis and bilateral thighs was also normal.

##### Diagnosis

The genetic sequencing of *AHCY* confirmed the familial homozygous pathogenic missense variant identified in his sister.

##### Therapeutic management

A methionine‐restricted diet was initiated at 14 months of age and has been followed for 2 years; however, adherence has been a challenge. In a more specific phase, the patient's protein intake, which included contributions from food and breastfeeds, was initially estimated at 28–31 g per day (2.8–3.1 g/kg/day). Over the course of 7 months, this intake was further reduced to 1.6 g/kg/day, equivalent to 32 mg methionine/kg/day. When the patient was 12 months old, the protein intake was adjusted to 1.3 g/kg/day. This level was above the safe level recommended for their age, which is 0.97 g/kg/day according to the FAO/WHO/UNU 2007 guidelines. A notable challenge in this process was the difficulty in accurately assessing the protein contribution from breastfeeds. This factor potentially caused variations in the total protein intake during the period. A methionine‐free amino acid supplement was introduced to complement the dietary protein and ensure a balanced nutritional intake. This supplement aimed to provide adequate levels of other essential amino acids, vitamins, and minerals. However, the patient showed an initial and on‐going dislike for the taste of the supplement. He only began to consistently consume it after 9 months on diet and then managed to continue for about 7 months at an approximate intake of 1.2 g/kg/day. Unfortunately, following a vomiting illness, the patient completely refused the supplement.

The dietary regimen was closely monitored through regular plasma or blood spot methionine measurements (Figure 4). However, taking these monthly blood spots for methionine monitoring presented increasing challenges for the parents and was not always feasible.

The family opted against therapy with creatine, phosphatidylcholine, and N‐acetylcysteine.

##### Evolution

Adherence to the diet has been a challenge. He remained clinically stable with low normal methionine levels 12–17 μmol/L (normal 10–60) (Figure 4, graph A). However, SAM and SAH levels, CK, and clotting remain persistently elevated. Liver transaminases responded partially (Figure 4, graphs B–G).

## 4 DISCUSSION

Here we expand the description of the mild phenotype of SAHHD with the report of two siblings with no evident clinical symptoms but biological evidence of high CK and liver dysfunction, reversible white matter changes, and subtle hypotrophic changes of the muscle MRI. These two patients of Pakistani origin have the same genotype, homozygous for the c.146G>A (p.Arg49His) pathogenic variant of the *AHCY* gene reported in two other Pakistani cases published by Stender et al. in 2015,^14^ presenting similarly with an asymptomatic presentation in childhood. One female patient presented with myopathic features in her mid‐twenties and a fatal hepatocellular carcinoma (HCC) at 32 years old. Her clinically asymptomatic son was diagnosed at 7 years old, with the same condition. These findings support a phenotype–genotype correlation and suggest a potential hotspot variant in the Asian population, notably from Pakistan. While the global population allele frequency for this variant is approximately 0.000012 (or 1/83 400), it is notably more common in South Asia with an allele frequency of approximately 0.000065 (or 1/15 300), aligning with the ethnicity of the cases we reported.^17^ Importantly, this mutation has been identified in other patients with SAHH deficiency, including some cases detailed in the paper by Stender et al.^14^ mild SAHHD is likely underdiagnosed, especially in South Asia as cases may remain clinically asymptomatic until early adulthood.

Only 16 SAHHD cases have been reported with a broad clinical spectrum,^18^, ^19^, ^20^ including different ages of onset, organ involvement, and disease severity.^7^, ^8^, ^9^, ^10^, ^11^, ^12^, ^13^, ^14^, ^15^, ^16^, ^18^, ^21^, ^22^ An important number of patients have severe encephalomyopathy, developmental delay, and profound hypotonia, causing fatal respiratory failure in infancy.^7^, ^8^, ^10^, ^11^, ^12^, ^13^, ^15^, ^16^, ^18^, ^20^, ^21^, ^22^ Milder patients present with non‐life‐threatening phenotypes in early infancy with chronic muscle and cognitive issues, have been described in the literature by both Buist et al.^9^ and Stender et al.^14^ (Table 1). For instance, while the patient reported by Buist et al. presented symptoms in the newborn period, including chronic muscle problems and significant psychiatric manifestations, they did not face severe life‐threatening symptoms within the initial months of life. This profile aligns more closely with the Croatian patients described, who likewise exhibited chronic muscle issues and mild cognitive impairments. The oldest living patient, initially diagnosed with undefined isolated hypermethioninemia through newborn screening, was confirmed to have SAHH deficiency, later, at 26 years old. Clinically, this individual was experiencing developmental delay and severe myopathy.^9^, ^23^ This rare condition is likely underdiagnosed. Isolated hypermethioninemia is an important biomarker to guide toward diagnosis. Unfortunately, newborn screening for elevated methionine is not reliable for detecting SAHHD.^1^, ^16^ As reported previously, markedly high methionine could be absent in many cases during the first weeks of life.^1^, ^15^, ^16^, ^20^, ^23^ Targeting SAH in dried blood spot is a challenge due to limited stability.^23^

**TABLE 1:** Summary of biochemical profiles at initial evaluation and clinical features before and after treatment in patients with a mild phenotype of SAH deficiency.

					Before therapy							Under therapy					
Patient	Gender	Ethnicity	Age at diagnosis	AHCY gene	Methionine (μmol/L)	CK (U/L)	ALT (IU/L)	SAM (nmol/L)	SAH (nmol/L)	Clinical features	Treatment	Methionine (μmol/L)	CK (U/L)	ALT (IU/L)	SAM (nmol/L)	SAH (nmol/L)	Clinical features
1^8^	M	USA (unreported ethnicity)	26 yo	Y143C/A89V	135–577	1400–3040	166	1933	772	Hypotonia and mild DD	Methionine‐restricted diet started at 5 M, stopped at 6 yo	10–60	1400–3500	69	1794	532	Generalized muscle weakness, IQ 64 at age 20
2^13^	M	Pakistani	7 yo	Arg49H/Arg49H	528	N/A	167	1930	3260	Asymptomatic	Methionine‐restricted diet started at 7 yo	10	N/A	31	162	133	Asymptomatic
3^13^	F	Pakistani	32 yo	Arg49H/Arg49H	N/A	1256	107	N/A	N/A	Learning disability; muscle weakness in her mid‐20s; HCC at 29 yo	Liver transplantation at 30 yo	N/A	N/A	N/A	N/A	N/A	Severe muscle weakness; died after transplantation at age 32
4^a^	F	Pakistani	6 yo	Arg49H /Arg49H	985	1450	250	2426	1408	Isolated weak verbal reasoning; mild diffuse leukodystrophy	Methionine‐restricted diet	20–100	571–5110	144–296	3670–3690	3060–6230	Normalization of brain MRI, subtle atrophy in the muscle MRI
5^a^	M	Pakistani	18 mo	Arg49H /Arg49H	384	1125	250	2730	2550	Asymptomatic	Methionine‐restricted diet	10–31	1282–2504	94–228	1140	6250	Asymptomatic

Abbreviations: ALT, alanine aminotransferase; CK, creatine kinase; DD, developmental delay; F, female; M, male; mo, months; N/A, not available/not applicable; SAH, S‐adenosylhomocysteine; SAM, S‐adenosylmethionine; yo, years.

^a^ This study.

Myopathy is a well‐recognized permanent feature of the disease. Ramadža et al. showed elevated muscle lipid fraction more prominent in the proximal skeletal muscles of the lower extremities by muscle MRI in three myopathic brothers with severe SAHHD.^18^ Neither of our patients showed clinical myopathy, but both had elevated CK at 5–10 times normal values. The muscle MRI of patient 1 showed subtle bilateral atrophy of the posterior compartment muscles, mainly in the adductor magnus, involving the same group of muscles described by Ramadža et al.^18^ This subclinical alteration highlights the slowly progressive muscle disease over time.

In SAHHD, methionine, SAM, and SAH accumulate upstream of the enzymatic block.^7^, ^8^, ^9^, ^19^ The pathophysiology remains unexplained mainly, although the molar concentration ratio of SAM/SAH, which regulates multiple methyltransferase reactions, is suggested to be the primary disease‐causing mechanism.^16^ A methionine‐restricted diet can decrease SAM and SAH levels.^7^, ^8^, ^10^, ^12^, ^13^, ^15^, ^16^ However, an effective reduction of SAM/SAH remains a significant challenge in clinical nutrition and metabolic management. The complexity of this issue lies partly in the current limited understanding of the mechanisms needed to effectively lower these compounds. Hypomethioninemia through dietary restrictions does not seem the best option and is challenging to achieve. Excessively lowering methionine intake can inadvertently affect the healthy growth and development of pediatric patients. In the literature, dietary management increased muscle strength, but all patients continued to experience developmental delay.^7^, ^8^, ^10^, ^14^, ^16^ Interestingly, the central nervous system seems to respond better to dietary therapy than other organs, for example, skeletal muscle and liver. For example, the most common neuroimaging findings in the literature were abnormal myelination and white matter atrophy.^7^, ^8^, ^10^, ^12^, ^15^, ^18^, ^22^ Some improvement was occasionally noted after starting dietary treatment,^8^, ^9^, ^18^ which we also observed in patient 1 with reversible white matter changes after only 12 months of dietary methionine restriction. This was achievable with lowering of methionine, but not to normal reference ranges. In parallel, SAM and SAH remained persistently high, likely due to their intracellular nature and the fact that their presence in plasma may result from cellular leakage. Our study supports previous observations of the rapid neurological benefit of a methionine‐restricted diet. However, it is important to note that while plasma levels of SAM and SAH did not normalize, it is possible that intracellular levels in organs such as the brain and muscle might be reduced with dietary restriction. Further studies, particularly in SAHHD animal models, are needed to explore the effects of methionine restriction on intracellular SAM and SAH levels.

Similar to published cases, our dietary intervention led to a decrease in ALT levels, though this did not normalize, while CK levels remained unchanged.^7^, ^8^, ^10^, ^16^ From our observations, it remains uncertain whether the course of the skeletal muscle and liver disease can be significantly modified by methionine restriction. The effect may vary depending on how low the plasma methionine level is and the residual enzyme activity. The fatal HCC diagnosis in a patient with mild SAHHD in her mid‐thirties warrants a long‐term follow‐up.^14^ There is reasonable evidence to associate SAHHD with increased liver tumorigenic risk, as abnormal methylation patterns are regularly observed in human cirrhosis and HCC cases.^24^, ^25^, ^26^ It is important to note that high methionine levels alone, as seen in conditions like MAT deficiency, do not typically result in cirrhosis or HCC.

Additional therapies have been considered. Various reports suggest the clinical benefit of phosphatidylcholine and N‐acetylcysteine supplementation,^1^, ^7^, ^9^, ^10^, ^12^, ^13^, ^15^, ^16^ alongside creatine, which the parents declined in our study due to the absence of clinical symptoms, impact on quality of life and lack of evidence of long‐term benefit. The inefficacy of dietetic intervention in decreasing SAH and SAM levels and the potential link with liver tumors raises the question of more invasive intervention, such as liver transplantation, even in milder forms of the disease.^15^ The long‐term clinical benefits of a methionine‐restricted diet on liver disease are not completely known.

In conclusion, we expand the description of the mild spectrum of SAHH deficiency with two novel patients with no apparent clinical symptoms. Our data support a genotype–phenotype correlation for the pathogenic *AHCY* variant R49H and a possible hotspot in the South Asian population. Methionine‐restricted diet reverses the brain white matter changes observed in isolated hypermethioninemia, though it remains unclear whether these changes are solely due to hypermethioninemia or whether additional pathophysiological mechanism could be involved. Regardless, the diet showed no identifiable benefits on skeletal muscle. This highlights the different responses of organs to methionine restriction in this rare inherited metabolic disease and raises the question of more invasive therapies, such as liver transplantation for SAHH deficiency.

## AUTHOR CONTRIBUTIONS

All authors reviewed and approved the final version of the manuscript.

## FUNDING INFORMATION

This study was supported by the NIHR Great Ormond Street Hospital Biomedical Research Centre MR/T008024/1 (to J. B.). The views expressed are those of the author(s) and not necessarily those of the NHS, the NIHR, or the Department of Health.

## CONFLICT OF INTEREST STATEMENT

The authors declare no conflicts of interest.

## ETHICS STATEMENT

All procedures followed were in accordance with the ethical standards of the responsible committee on human experimentation (institutional and national) and with the Helsinki Declaration of 1975, as revised in 2000. Participants' data were recorded anonymously. Informed consent was approved by the National Research Ethics Service Committee London‐Bloomsbury (13/LO/0168) and written consents for publication were obtained from both patients from their parents.

## CONSENT TO PARTICIPATE

This research study was conducted retrospectively from medical notes. Participants' data were recorded anonymously. Informed consent was approved by the National Research Ethics Service Committee London‐Bloomsbury (13/LO/0168) and written consents for publication were obtained from both patients from their parents.

## Data Availability

The data used in this study are available from the corresponding author upon reasonable request.
